# Inflammatory cytokines and chemokines in obese adolescents with antibody against to adenovirus 36

**DOI:** 10.1038/s41598-023-33084-4

**Published:** 2023-06-19

**Authors:** Marcelo D’Alessandre Sanches, Tamara Beres Lederer Goldberg, Anapaula da Conceição Bisi Rizzo, Valéria Nóbrega da Silva, Luciana Nunes Mosca, Graziela Gorete Romagnoli, Carolina Mendonça Gorgulho, João Pessoa Araujo Junior, Gustavo Ramos de Lima, Isabella Rodrigues Betti, Cilmery Suemi Kurokawa

**Affiliations:** 1 Medical School, São Paulo State University (Unesp), Botucatu, Brazil; 2 Institute of Biosciences, São Paulo State University (Unesp), Botucatu, Brazil

**Keywords:** Immunology, Microbiology, Diseases

## Abstract

Obesity in adolescents has reached epidemic proportions and is associated with the inflammatory response and viral infections. The aim of this study was to understand the profile of inflammatory cytokines and chemokines associated with the inflammatory response and metabolic syndrome (MetS) in obese adolescents with positive serology for adenovirus 36 (ADV36). Thirty-six overweight, 36 obese, and 25 severe obesity adolescents aged 10 to 16 years were included in the study. The following variables were analyzed: sex, age, body mass index (BMI), blood pressure, total cholesterol and fractions, triglycerides, glucose, serum cytokine concentrations, and ADV36 antibodies. Cytokines and chemokines were quantified by cytometry and ADV36 serology was determined by enzyme-linked immunosorbent assay (ELISA). The results showed higher levels of the cytokines interleukin-1beta (IL-1β), IL-6, IL-10 and of the chemokine interferon-gamma-inducible protein 10 (IP-10) in severe obesity adolescents compared to the obese and overweight groups, as well as in the group with MetS compared to the group without this syndrome. The frequency of ADV36-positive individuals did not differ between groups. The findings revealed differences in BMI between the obese and severe obesity groups versus the overweight group in the presence of positivity for ADV36, suggesting an association with weight gain and possibly MetS installation.

## Introduction

Obesity in children and adolescents has acquired alarming and epidemic proportions in recent decades on several continents^1–3^ and is considered a serious public and social health problem. Since 2015, obesity has been recognized as a “disease” and no longer as a complicating factor associated with another disease^4^. This fact has led to an increase in the incidence and prevalence of comorbidities and diseases in children, adolescents, and adults^3, 5–7^.

The high prevalence of obesity in adolescents is associated with an increased risk of bone alterations/wear-and-tear, cancer, type 2 diabetes mellitus, arterial hypertension, heart disease, stroke, increased risk of early mortality, and metabolic syndrome (MetS)^7–12^. Identifying MetS in obese adolescents is important to suggest early interventions in diet, lifestyle and therapy when necessary^13^, with the prevention of the consequences of excessive weight gain that seems to occur at increasingly earlier ages^5^. Metabolic syndrome consists of a set of metabolic alterations and is recognized as a risk factor for the development of cardiovascular diseases and type 2 diabetes mellitus^14^. The determinant factors of this syndrome are obesity, hypertension, dyslipidemia, and hyperglycemia^15^.

The origin of the complications associated with obesity lies in the systemic and chronic inflammation of adipose tissue^16–18^. The production of inflammatory cytokines is generally associated with a certain subpopulation of macrophages, called classically activated or M1 macrophages, which participate in the pathophysiology of obesity^19^. Macrophages are the main cells that produce the inflammatory cytokines IL-1β, IL-6, IL-8, IL-10, IL-12, and tumor necrosis factor-alpha (TNF-α), as well as chemokines that also seem to be produced by adipose tissue in obesity^20, 21^. The chemokines produced by macrophages/monocytes and linked to obesity are IP-10 or chemokine (C-X-C motif) ligand 10 (CXCL10), which is associated with decreased angiogenesis in adipose tissue and inflammation due to the lack of vascularization and is expressed in morbidly obese patients^22^, and regulated on activation, normal T cell expressed and secreted (RANTES) or chemokine (C–C motif) ligand 5 (CCL5), which contributes to resistance to insulin^23, 24^. Other chemokines that are being investigated in obese individuals and that have been associated with inflammation in adipose tissue are monocyte chemoattractant protein-1 (MCP-1) or CCL2 and monokine induced by interferon-gamma (MIG) or CXCL9^20, 25^.

It is also believed that obesity has an infectious component, i.e., some viral infections may play a role in weight gain and obesity. Within this context, several studies have linked obesity to infection with adenovirus 36 (ADV36), a virus already described in adults, adolescents, and children^26, 27^. This adenovirus interferes with insulin resistance and cytokine production in obese individuals. Some studies reported the presence of ADV36 in adipose tissue removed from obese patients, reinforcing the association of this adenovirus infection with obesity^28^.

Within this context, the aim of the present study was to identify and quantify serum concentrations of inflammatory cytokines and chemokines associated with the inflammatory profile of M1 macrophages, as well as to evaluate associations with the grade of obesity, positive ADV serology, and presence of MetS in adolescents.

## Results

### Anthropometric data and biochemical assessment for the characterization of MetS

Ninety-seven patients were included. These patients were divided into three groups according to BMI (Table 1). Some data on sexual maturation were not obtained due to the patient’s refusal during the evaluation. All patients were submitted to biochemical assessment for the identification of MetS and characterization of the groups (Table 1).

**Table 1:** Mean and standard deviation of the anthropometric, clinical and biochemical data of overweight, obese and severe obesity adolescents.

Mean ± SD	Overweight (n = 36)		Obese (n = 36)		Severe obesity (n = 25)	
Age (years)	13.96 ± 2.20		13.39 ± 2.34		13.29 ± 1.99	
BMI (kg/m^2^)	24.22 ± 1.63		28.58 ± 2.79		33.96 ± 4.18	
SBP (mmHg)	112.15 ± 13.03		118.13 ± 14.99		124.00 ± 16.62	
DBP (mmHg)	71.33 ± 8.68		74.27 ± 9.39		77.65 ± 12.01	
Total cholesterol (mg/dL)	156.88 ± 22.74		155.36 ± 32.83		173.68 ± 32.98	
Triglycerides (mg/dL)	107.25 ± 43.82		114.44 ± 53.32		137.08 ± 61.11	
Glycemia (mg/dL)	85.22 ± 8.39		83.03 ± 7.19		85.32 ± 7.35	
HOMA-IR	3.21 ± 2.17		3.54 ± 1.90		5.42 ± 3.64	
QUICKI	0.33 ± 0.02		0.32 ± 0.02		0.30 ± 0.02	
Male sex (frequency)	15/36		12/36		10/25	
Sexual maturation, Tanner^29^ criteria (frequency)	Male	Female	Male	Female	Male	Female
1–2	0	1	1	0	1	1
3–4	7	5	8	12	7	7
5	2	8	2	7	0	5

*SD* standard deviation, *BMI* body mass index, *SBP* systolic blood pressure, *DBP* diastolic blood pressure, *HOMA-IR* homeostasis model assessment of insulin resistance, *QUICKI* quantitative insulin sensitivity check index.

### Inflammatory cytokines IL-1β, IL-6 and IL-10 associated with MetS

**Figure 1:**
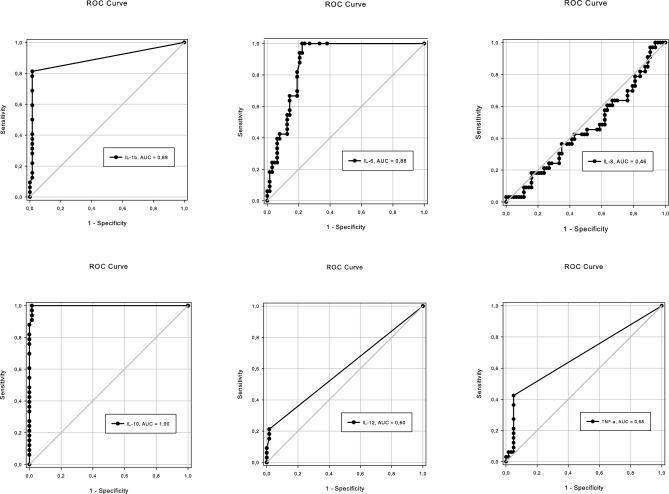
ROC curve of cytokines. Analysis of IL-1β, IL-6, IL-8, IL-10, IL-12p40, and TNF-α in serum of adolescents with excess weight. AUC = area under the curve indicating the accuracy of each cytokine as a diagnostic marker of MetS in overweight and obese adolescents.

Cytokines were measured in all groups. Differences between the overweight, obese and severe obesity groups were observed for IL-1β, IL-6, and IL-10 (Table 2). The levels of these cytokines differed between the groups without and with MetS (Table 3). The areas under the receiver operating characteristics (ROC) curve were also higher for these cytokines, with values ranging from 0.88 to 1.0 (Fig. 1).

**Table 2:** Cytokines in overweight, obese and severe obesity adolescents.

Median (p25; p75)	Overweight (n = 36)	Obese (n = 36)	Severe obesity (n = 25)	*p*
IL-1β	0.01 (0.01;0.01)a	0.01 (0.01;0.42)ab	0.18 (0.01;1.11)b	0.026
IL-6	0.09 (0.01;2.49)a	0.82 (0.01;2.17)a	3.65 (0.37;5.89)b	0.010
IL-8	10.76 (7.62;16.78)	9.72 (7.29;15.54)	8.22 (5.52;11.99)	0.299
IL-10	0.01 (0.01;0.01)a	0.01 (0.00;1.55)a	1.74 (0.01;2.55)b	0.002
IL-12	0.01 (0.01;0.01)	0.01 (0.01;0.01)	0.01 (0.01;0.01)	0.669
TNF-α	0.01 (0.01;0.01)	0.01 (0.01;0.01)	0.01 (0.01;0.01)	0.922

p25 = 25th percentile; p75 = 75th percentile; *p* = significance. Different letters indicate a significant difference between the groups analyzed. Kruskal–Wallis test.

**Table 3:** Cytokines and chemokines in adolescents with excess weight without and with MetS using four risk factors for the definition of MetS.

Median (p25; p75)	No MetS (n = 63)	With MetS (n = 33)	*p*
IL-1β	0.00 (0.00;0.00)	1.00 (0.36;1.66)	< 0.001
IL-6	0.00 (0.00;0.98)	3.17 (2.17;6.46)	< 0.050
IL-8	10.29 (7.62;15.41)	7.56 (4.07;11.26)	ns
IL-10	0.00 (0.00;0.00)	2.10 (1.72;2.59)	< 0.001
IL-12	0.00 (0.00;0.00)	0.00 (0.00;0.00)	ns
TNF-α	0.00 (0.00;0.00)	0.00 (0.00;0.64)	ns
IP-10	7.56 (4.07;11.26)	183.96 (118.81;248.91)	< 0.001
MCP-1	117.45 (78.10;161.76)	123.93 (92.45;160.09)	0.683
MIG	78.87 (57.31;102.25)	71.85 (57.85;92.02)	0.403

p25 = 25th percentile; p75 = 75th percentile; *p* = significance; ns = not significant. Mann–Whitney test.

### Chemokine IP-10 associated with MetS

**Figure 2:**
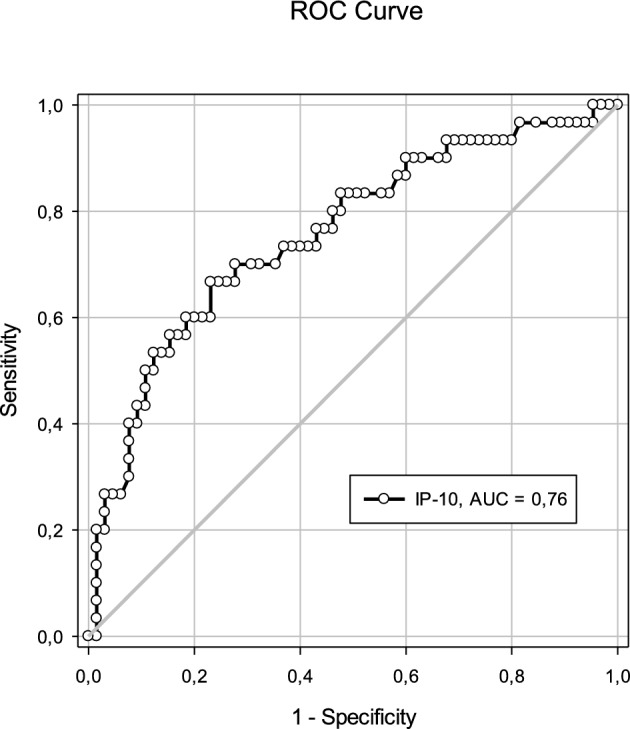
ROC curve of chemokine IP-10. Analysis of IP-10 in serum of adolescents with excess weight. AUC = area under the curve indicating the accuracy of the chemokine as a diagnostic marker of MetS in overweight and obese adolescents. Cut-off = 150.36 pg/mL; specificity = 0.28; sensitivity = 0.70.

Chemokines were measured in all groups. There were differences in IP-10 levels between the severe obesity groups and the other groups, while the overweight group did not differ from the obese group for any of the chemokines evaluated (Table 4). A difference in IP-10 was observed between the groups without and with MetS (Table 3). The ROC curve for IP-10 showed an accuracy of 0.76, with a cut-off value of 150.36 pg/mL (Fig. 2). There was no significant difference in the other chemokines between groups.

**Table 4:** Chemokines in overweight, obese and severe obesity adolescents.

Median (p25; p75)	Overweight (n = 36)	Obese (n = 36)	Severe obesity (n = 25)	*p*
IP-10	117.00 (77.00;185.00)a	118.00 (77.00;170.00)a	166.00 (139.00;226.00)b	0.050
MCP-1	119.00 (103.00;181.00)	119.00 (82.00;146.00)	92.00 (74.00;148.00)	0.220
MIG	87.00 (56.00;99.00)	75.00 (60.00;5.00)	73.00 (57.00;107.00)	0.770

p25 = 25th percentile; p75 = 75th percentile; *p* = significance. Different letters indicate a significant difference between the groups analyzed. Kruskal–Wallis test.

### Anti-ADV36 antibody in the overweigh, obese and severe obesity groups

There was no difference in positivity for ADV36 between the degrees of obesity, presence of MetS and association with levels of cytokines IL-1β, IL-6 and IL-10 (Table 5). The evaluation of BMI in the ADV36 positive group separated BMI into only two groups, that is, BMI of obese individuals was equal to BMI of severe obesity individuals and both were higher than the group of overweight adolescents (Table 6). This difference in BMI in two groups did not occur when adolescents who were negative for ADV36 were evaluated; this comparison showed overweight BMI < obese BMI < severe obesity BMI (Table 6).

**Table 5:** Number of individuals with positive anti-ADV36 serology.

	Overweight (n = 36)	Obese (n = 36)	Severe obesity (n = 25)	*p*
Subjects positive for ADV36	14	18	5	0.060
Percentage	39	50	20	ns
♂ ADV36+/total ♂	4/15	2/12	1/10	0.438
♂ ADV36+/total ADV36+	4/14	2/18	1/5	0.456
MetS ADV36+	1	4	2	ns
IL-1β (median; min.; max.)	(0.01;0.01;0.79)a	(0.01;0.01;1.66)ab	(0.36;0.01;3.59)b	0.026
IL-6 (median; min.; max.)	(0.04;0.01;14.44)a	(0.01;0.01;24.49)a	(3.18;0.01;7.04)b	0.010
IL-10 (median; min.; max.)	(0.01;0.01;2.10)a	(0.01;0.01;2.70)a	(2.28;0.01;3.59)b	0.023
IP-10 (median; min.; max.)	(116.00;42.00;701.00)a	(118.00;20.00;374.00)a	(166.00;86.00;369.00)b	0.050

min. = minimum; max. = maximum; *p* = significance; ns = not significant. Different letters indicate a significant difference between the groups analyzed. Kruskal–Wallis test for comparison of medians. Chi-square test or Fisher's exact test to compare the frequency between groups.

**Table 6:** BMI of overweight, obese and severe obesity adolescents with positive or negative anti-ADV36 serology.

Median (p25; p75)	Overweight (n = 36)	Obese (n = 36)	Severe obesity (n = 25)	*p*
ADV36+	24.53 (23.48;25.53)aA	29.21 (25.95;31.93)bA	38.76 (28.71;39.70)bA	≤ 0.050
ADV36−	24.35 (23.21;25.07)aA	27.32 (26.65;30.12)bA	33.02 (31.28;36.38)cA	≤ 0.050
p	> 0.050	> 0.050	> 0.050	

p25 = 25th percentile; p75 = 75th percentile; p = significance. Lowercase letters: comparison between grades of obesity. Uppercase letters: intragroup comparison of the presence and absence of ADV36 in the overweight, obese and severe obesity groups. Different letters indicate a significant difference between the groups analyzed. Kruskal–Wallis test for comparison of medians. *t* test for intragroup comparison of the presence and absence of ADV36 in the overweight, obese and severe obesity groups.

## Discussion

The results revealed differences in inflammatory and anti-inflammatory cytokines between the groups studied, as demonstrated by the difference in the levels of IL-1β, IL-6 and IL-10, cytokines produced by M1 and M2 macrophages. The group of adolescents with excess weight had higher levels than those with lower weight and BMI, suggesting the potential use of these cytokines as diagnostic markers of comorbidities and MetS in obese adolescents. Associations of inflammatory cytokines with obesity and BMI and their participation in the pathophysiology of weight gain are frequently reported. Therefore, obesity is referred to as a state of low-grade chronic metabolic inflammation or meta-inflammation^16, 17^.

Meta-inflammation involves the participation of immune response cells, especially activated monocytes and macrophages. These cells infiltrate adipose tissue where they are transformed to macrophages or adipose tissue macrophages, inducing the maturation and expansion of adipose tissue and an increase in the size of fat cells. Adipose tissue macrophages can be classified as M1 macrophages, which produce IL-6, TNF-α, MCP-1, CD11c, and inducible nitric oxide synthase (iNOS), and M2 macrophages, which produce IL-10, transforming growth factor beta 1 (TGF-β1) and arginine^30^.

IL-1β, which is produced and detected in meta-inflammation of obesity, alters the production of inflammatory cytokines and chemokines in pre-adipocytes. Pre-adipocytes incubated with IL-1β release chemokines and inflammatory cytokines within a period of 24 h. An increase could also be detected by quantitative polymerase chain reaction after 4 h of incubation. IL-1β stimulates the secretion of IL-6, IL-8, IL-10, IL-13, MCP-4, TNF-α and IP-10 by monocytes, macrophages, preadipocytes, and adipocytes. These cells seem to be sensitive to the action of and exposure to IL-1β, inducing the secretion of inflammatory cytokines. These facts suggest that elevation of this cytokine may be a trigger for the production of inflammatory cytokines and chemokines associated with vascular and tissue damage in obesity^31, 32^.

IL-6 is secreted by M1 macrophages, adipocytes, and fibroblasts. This production is associated with exposure to high levels of glucose^33^. In addition to these effects, IL-6 seems to regulate body weight, lipid metabolism^34, 35^, insulin resistance^36–38^, and the production of C-reactive protein. Stating that IL-6 is a cause or a consequence of meta-inflammation is still controversial but some studies have demonstrated that adipocytes respond positively to this cytokine or produce it. Obesity upregulates IL-6 expression and IL-6 receptor (IL-6R) in adipose tissue and that this production contributes to the meta-inflammation in obesity^37, 39^.

Macrophages are sources of IL-10 in overweight inflammation. This cytokine plays a role in the containment of the immune response, modulating and interfering with the innate and adaptive response. The increase in IL-10 is cited as an independent factor of nonalcoholic fatty liver disease (NAFLD) in obese children, suggesting that this cytokine is a protective factor against NAFLD^40^. A study investigating the participation of IL-10 in an experimental model of steatosis suggested that CCR7+ mononuclear cells in the liver could regulate obesity-induced hepatic steatosis via the induction of IL-10-expressing invariant natural killer T cells^41^.

Cytokines in general may serve as auxiliary tools in the diagnosis of MetS but should not be employed individually. These mediators of innate immunity can complement clinical and laboratory parameters already documented in obese adolescents. It is important to point out that, although obesity is characterized by a state of inflammation, not all cytokines are detectable in serum. The present study analyzed cytokines produced by M1 macrophages, the target cells in obesity studies. Obese individuals with metabolic problems exhibit an increase in TNF-α, IL-6 and IL-1β accompanied by a decrease in adiponectin^42^. Several studies have suggested an association with the presence of M1 macrophages and metabolic disorder.

The use of these cytokines as a diagnostic method must be interpreted with caution. In general, the accuracy values obtained in the present study for the cytokines are considered good (0.80 to 0.89) but not excellent (> 0.90)^43^. However, these cytokines certainly reflect the inflammatory state of the individual with excess weight and can be used to monitor the presence of inflammation which, in turn, causes tissue and vascular damage and induces metabolic changes.

The evaluation of MCP-1 and MIG chemokine levels did not differ in the groups studied, except for IP-10. The levels of IP-10 were higher in the severe obesity group and the accuracy obtained for this chemokine was 0.8. Among obese individuals, elevated serum IP-10 levels have been observed in patients with heart problems compared to those without such problems and have been associated with left ventricular failure and possible unsuccessful cardiac remodeling^44, 45^. This chemokine is secreted by macrophages and monocytes after stimulation with interferon gamma (IFN-γ) and is chemotactic for monocytes/macrophages, T cells, natural killer cells, and dendritic cells. In addition to these associations, IP-10 induces the proliferation of vascular smooth muscle cells, suggesting its participation in the onset and aggravation of atherosclerosis^46^.

Obesity is a chronic inflammatory state that is characterized by the presence of inflammatory and anti-inflammatory cytokines and chemokines produced by M1 macrophages. Elucidating their role in the pathophysiology of obesity and its comorbidities may permit to identify intracellular activation pathways and therapeutic targets^47^. The chemokines MCP-1, IP-10 and IL-8 have been indicated as therapeutic targets for hypertension because of their participation in atherosclerosis, myocardial complications, and renal fibrosis^48^.

Regarding the presence of ADV36, this evaluation was not significant in the comparison between the groups of obesity and positivity for ADV36, but there was a difference in the evaluation within the severe obesity group, with a higher frequency of severe obesity adolescents positive for ADV36. The presence of infection is related to increased BMI (Table 6).

The association of the presence of ADV36 with obesity has been described since 2000 in adults^49^ and since 2010 in children^50^. ADV36 induces differentiation or adipogenesis of somatic cells to adipocytes. This change is mediated by incorporation of the viral gene early gene 4, open reading frame 1 (E4-ORF1) in the cell nucleus, which accelerates adipogenesis and induces the production of inflammatory and anti-inflammatory cytokines and adipokines. The increase in adipocytokines such as MCP-1, TNF-α, IL-1, and IL-6 causes an increase in fat nodules and alters fat metabolism during the inflammatory process. M1 macrophages appear to play a key role in cytokine production and adipose tissue inflammation^26, 51–54^.

In a study of 291 children, Berger et al*.*^55^ found an increase in TNF-α and IL-6 in ADV36-positive children. The odds ratio for TNF-α and IL-6 was 2.2 and 2.4, respectively, in ADV36-positive children aged 9–13 years. In addition to these data, some experimental studies using mouse macrophages have shown that ADV36 induces the production of MCP-1 in vitro^53^.

In conclusion, IL-1β, IL6, IL-10, and IP-10 levels are elevated in adolescents with severe obesity and these cytokines can serve as a tool for the diagnosis of MetS. As for the limitations of the study, it was not possible to make a food survey, due to the inability to keep adolescents under surveillance of food eaten for 3 days and associate the type of food with inflammation. Regarding the presence of ADV36 infection, there was an increase in BMI in the obese group, bringing these indices closer to the severe obesity group, suggesting that ADV36 causes aggravation of weight gain. A larger sample is needed to confirm the association of infection with BMI gain. The correlation of infection with sex and MetS did not occur, despite the higher frequency in girls.

## Methods

### Study design

Cross-sectional study of male and female adolescents aged 10 to 16 complete years, who were seen at the Botucatu Medical School, Unesp, from September 2018 to September 2019, and who did not use medications. Participants were consecutively included and allocated to the groups according to the degree of obesity. The sample consisted of 97 adolescents, including 36 overweight, 36 obese and 25 severe obesity individuals.

### Ethics declaration

The Ethics Committee on Research Involving Humans of the Botucatu Medical School, Unesp, São Paulo, Brazil, approved the study on November 9, 2020 (Approval number 4.399.388). All methods were performed in accordance with the relevant guidelines and regulations. All adolescents and legal representatives signed the free informed consent form for inclusion in the study.

### Clinical variables

Weight and height were obtained according to the National Health and Nutrition Examination Survey^56^.

The nutritional status was evaluated using age- and sex-specific BMI [weight (kg)/height^2^ (m)] curves and the respective cut-off points proposed by Kuczmarski et al*.*^56^. Overweight, obesity and severe obesity were defined according to Kuczmarski et al*.*^56^ and Freedman et al*.*^57^. The following sex- and age-specific definitions were adopted: overweight, 85th ≥ BMI < 95th; obese, 95th ≥ BMI ≤ 99th; severe obesity, BMI > 99th.

Waist circumference was measured as described by Rizzo et al*.*^58^ and the values were compared to the values of the 90th percentile for abdominal circumference according to age and sex^59^.

The SBP and DBP were measured twice and the mean of these measurements was used for analysis^60^.

### Laboratory tests

To evaluate the presence of MetS criteria in the adolescents, the following laboratory parameters were measured in a Vitros 950 dry chemical analyzer (Johnson & Johnson): total cholesterol and fractions [high-density lipoprotein (HDL), low-density lipoprotein (LDL) and very low-density lipoprotein (VLDL)], triglycerides, and fasting glucose. The HOMA-IR [(fasting blood glucose mmol/L) × (fasting insulinemia µU/ml)/22.5]^61^ and QUICKI [1/log (fasting insulinemia µU/ml) + log (fasting blood glucose mg/dl)]^62^ values were calculated based on the fasting blood glucose and insulin measurements to identify probable insulin resistance. The measurements were performed in the morning after a 10-h fast.

### Definition of MetS in adolescents aged 10 to 16 years

MetS was defined based on the presence of central obesity using the respective waist circumference percentiles (≥ 90th percentile for sex and age), combined with three additional factors (elevated triglycerides, low HDL-cholesterol, arterial hypertension, and hyperglycemia). The following altered values were considered for both sexes: triglycerides ≥ 150 mg/dL, HDL-cholesterol < 40 mg/dL, arterial hypertension with SBP ≥ 130 mmHg or DBP ≥ 85 mmHg, and fasting glucose ≥ 100 mg/dL or a previous diagnosis of type 2 diabetes^15, 58^.

### Exclusion criteria

Adolescents with the presence of metabolic, endocrine or genetic disease verified by the history of the current disease, the general physical examination and laboratory or radiodiagnostic procedures, adolescents with diabetes mellitus, gastrointestinal disease, kidney disease, early or late puberty, current or past pregnancy, who used hormonal contraceptives, in corticosteroid therapy and in chronic use of medications, as well as adolescents who did not attend the commitments to measure clinical variables or laboratory tests were excluded from the study.

### Quantification of cytokines and chemokines by cytometry

Cytokines IL-1β, IL-6, IL-8, IL-10, IL-12, TNF-α and chemokines IP-10, MCP-1, MIG were quantified by ELISA using beads coated with monoclonal antibodies against each cytokine analyzed. Phycoerythrin (PE) was used as an antibody detector against each cytokine of interest.

Briefly, beads labeled with different fluorescence intensities were mixed in a tube at a rate of 10 µL/test of each bead coated with the specific anti-cytokine antibody and the procedures recommended by the manufacturer were followed. A volume of 50 µL of the bead mixture was transferred to the test tubes and reserved for later addition of the samples and recombinant standard. After this procedure, the PE-labeled detector antibody was added. Fluorescence of the labeled beads was acquired in a FACSCanto II cytometer (BD Biosciences, USA) and the data were analyzed using the FlowJo software, following the procedures of the manufacturer of the Human Inflammation and Human Chemokine kits (BD Biosciences, USA). A total of 1,800 or 300 events were counted for each bead or cytokine.

### Quantification of anti-ADV36 antibody in plasma

The anti-ADV36 antibody was analyzed qualitatively by ELISA using the MMBS 9310682 kit (MyBiosource, San Diego, CA, USA). A cut-off value of 0.194 was defined, i.e., samples with an optical density above the cut-off were defined as positive.

### Statistical analysis

The results were first analyzed by the Shapiro–Wilk test to verify the normality of the data. Parametric variables were compared between two groups by the unpaired *t* test and between three or more groups by one-way analysis of variance (ANOVA) followed by the Tukey test. Nonparametric variables were compared between groups using the Mann–Whitney test or Kruskal–Wallis test followed by Dunn’s test. ROC curves were constructed to evaluate the accuracy of the cytokines and the cut-off value of each cytokine was obtained from the inflection point of the curve. The chi-squared test or Fisher’s exact test was used to compare categorical data/frequencies. Data were analyzed using the SigmaPlot 12.0 for Windows statistical package (Jandel Corporation, CA, USA). Differences were considered significant when *p* < 0.05.

## Data Availability

All the data generated or analyzed during this study are included in this published article.

## References

[1] Bahia L (2012). The costs of overweight and obesity-related diseases in the Brazilian public health system: Cross-sectional study. BMC Public Health.

[2] Hales CM, Carroll MD, Fryar CD, Ogden CL (2017). Prevalence of obesity among adults and youth: United States, 2015–2016 key findings data from the national health and nutrition examination survey. NCHS Data Br.

[3] Weihrauch-Blüher S, Schwarz P, Klusmann JH (2019). Childhood obesity: increased risk for cardiometabolic disease and cancer in adulthood. Metabolism.

[4] Ponterio E, Gnessi L (2015). Adenovirus 36 and obesity: An overview. Viruses.

[5] Goodman E, Daniels SR, Meigs JB, Dolan LM (2007). Instability in the diagnosis of metabolic syndrome in adolescents. Circulation.

[6] Kumar S, Kelly AS (2017). Review of childhood obesity: From epidemiology, etiology, and comorbidities to clinical assessment and treatment. Mayo Clin. Proc..

[7] Fang X (2021). Causal association of childhood obesity with cancer risk in adulthood: A Mendelian randomization study. Int. J. Cancer.

[8] Ma Y (2012). Determinants of racial/ethnic disparities in incidence of diabetes in postmenopausal women in the U.S.: The women’s health initiative 1993–2009. Diabetes Care.

[9] Zhang H, Rodriguez-Monguio R (2012). Racial disparities in the risk of developing obesity-related diseases: A cross-sectional study. Ethn. Dis..

[10] Kitahara CM (2014). Association between class III obesity (BMI of 40–59 kg/m^2^) and mortality: A pooled analysis of 20 prospective studies. PLoS Med..

[11] Tanamas SK (2016). Quantifying the proportion of deaths due to body mass index- and waist circumference-defined obesity. Obesity.

[12] Weihe P, Spielmann J, Kielstein H, Henning-Klusmann J, Weihrauch-Blüher S (2020). Childhood obesity and cancer risk in adulthood. Curr. Obes. Rep..

[13] Cook S, Weitzman M, Auinger P, Nguyen M, Dietz WH (2003). Prevalence of a metabolic syndrome phenotype in adolescents findings from the third national health and nutrition examination survey, 1988–1994. Arch. Pediatr. Adolesc. Med..

[14] Fahed G (2022). Metabolic syndrome: Updates on pathophysiology and management in 2021. Int. J. Mol. Sci..

[15] Zimmet P (2007). The metabolic syndrome in children and adolescents: An IDF consensus report. Pediatr. Diabetes.

[16] Gregor MF, Hotamisligil GS (2011). Inflammatory mechanisms in obesity. Annu. Rev. Immunol..

[17] Lumeng CN, Saltiel AR (2011). Inflammatory links between obesity and metabolic disease. J. Clin. Invest..

[18] Guerreiro VA, Carvalho D, Freitas P (2022). Obesity, adipose tissue, and inflammation answered in questions. J. Obes..

[19] Martinez FO, Gordon S (2014). The M1 and M2 paradigm of macrophage activation: Time for reassessment. F1000Prime Rep..

[20] Kim CS (2006). Circulating levels of MCP-1 and IL-8 are elevated in human obese subjects and associated with obesity-related parameters. Int. J. Obes. (Lond.).

[21] Mikhailova SV, Ivanoshchuk DE (2021). Innate-immunity genes in obesity. J. Pers. Med..

[22] Hueso L (2018). Upregulation of angiostatic chemokines IP-10/CXCL10 and I-TAC/CXCL11 in human obesity and their implication for adipose tissue angiogenesis. Int. J. Obes. (Lond.).

[23] Chang CC (2015). Interferon gamma-induced protein 10 is associated with insulin resistance and incident diabetes in patients with nonalcoholic fatty liver disease. Sci. Rep..

[24] Chou SY (2016). CCL5/RANTES contributes to hypothalamic insulin signaling for systemic insulin responsiveness through CCR5. Sci. Rep..

[25] Harakeh S (2020). Chemokines and their association with body mass index among healthy Saudis. Saudi J. Biol. Sci..

[26] Genoni G (2014). Obesity and infection: Two sides of one coin. Eur. J. Pediatr..

[27] Marjani A (2022). Adenovirus 36 infection and obesity meta-analysis of community-based studies. Rev. Med. Virol..

[28] Esposito S, Preti V, Consolo S, Nazzari E, Principi N (2012). Adenovirus 36 infection and obesity. J. Clin. Virol..

[29] Tanner JM (1981). Growth and maturation during adolescence. Nutr. Rev..

[30] Michaud A, Pelletier M, Noël S, Bouchard C, Tchernof A (2013). Array and protein array studies demonstrate that abdominal adipose tissues. Obesity (Silver Spring).

[31] Alomar SY (2015). PCR array and protein array studies demonstrate that IL-1 *β* (interleukin-1 *β*) stimulates the expression and secretion of multiple cytokines and chemokines in human adipocytes. Arch. Physiol. Biochem..

[32] Alomar SY, Gentili A, Zaibi MS, Kępczyńska MA, Trayhurn P (2016). IL-1*β* (interleukin-1*β*) stimulates the production and release of multiple cytokines and chemokines by human preadipocytes. Arch. Physiol. Biochem..

[33] Sundararaj KP (2009). Interleukin-6 released from fibroblasts is essential for up-regulation of matrix metalloproteinase-1 expression by U937 macrophages in coculture: Cross-talking between fibroblasts and U937 macrophages exposed to high glucose. J. Biol. Chem..

[34] Mohamed-Ali V (1997). Subcutaneous adipose tissue releases interleukin-6, but not tumor necrosis factor-α, in vivo. J. Clin. Endocrinol. Metab..

[35] Shoelson SE, Lee J, Goldfine AB (2006). Inflammation and insulin resistance. Erratum in: J. Clin. Invest..

[36] Senn JJ, Klover PJ, Nowak IA, Mooney RA (2002). Interleukin-6 induces cellular insulin resistance in hepatocytes. Diabetes.

[37] Eder K, Baffy N, Falus A, Fulop AK (2009). The major inflammatory mediator interleukin-6 and obesity. Inflamm. Res..

[38] Schultz O (2010). Effects of inhibition of interleukin-6 signalling on insulin sensitivity and lipoprotein (A) levels in human subjects with rheumatoid diseases. PLoS ONE.

[39] Sindhu S (2015). Obesity is a positive modulator of IL-6R and IL-6 expression in the subcutaneous adipose tissue: Significance for metabolic inflammation. PLoS ONE.

[40] Shi J-Q, Shen W-X, Wang X-Z, Huang KE, Zou C-C (2017). Relationship between immune parameters and non-alcoholic fatty liver disease in obese children. Indian Pediatr..

[41] Kim HM (2018). iNKT cells prevent obesity-induced hepatic steatosis in mice in a C-C chemokine receptor 7-dependent manner. Int. J. Obes. (Lond.).

[42] Navarro E, Funtikova AN, Fíto M, Schröder H (2015). Can metabolically healthy obesity be explained by diet, genetics, and inflammation?. Mol. Nutr. Food Res..

[43] Polo TCF, Miot HA (2020). Use of roc curves in clinical and experimental studies. J. Vasc. Bras..

[44] Altara R (2015). Left ventricular dysfunction and CXCR3 ligands in hypertension: From animal experiments to a population-based pilot study. PLoS ONE.

[45] Altara R (2016). CXCL10 is a circulating inflammatory marker in patients with advanced heart failure: A pilot study. J. Cardiovasc. Transl. Res..

[46] Wang HJ (2017). IP-10/CXCR3 axis promotes the proliferation of vascular smooth muscle cells through ERK1/2/CREB signaling pathway. Cell Biochem. Biophys..

[47] Martos-Moreno GA, Kopchick JJ, Argente J (2013). Adipoquinas en el nin˜o sano y con obesidad. An. Pediatr. (Barc.).

[48] Martynowicz H, Janus A, Nowacki D, Mazur G (2014). The role of chemokines in hypertension. Adv. Clin. Exp. Med..

[49] Dhurandhar NV (2000). Increased adiposity in animals due to a human virus. Int. J. Obes. Relat. Metab. Disord..

[50] Almgren M (2012). Adenovirus-36 is associated with obesity in children and adults in Sweden as determined by rapid ELISA. PLoS ONE.

[51] Pasarica M (2008). Adipogenic human adenovirus Ad-36 induces commitment, differentiation, and lipid accumulation in human adipose-derived stem cells. Stem Cells.

[52] Rogers PM (2008). Human adenovirus Ad-36 induces adipogenesis via its E4 orf-1 gene. Int. J. Obes. (Lond.).

[53] Na HN, Nam JH (2012). Adenovirus 36 as an obesity agent maintains the obesity state by increasing MCP-1 and inducing inflammation. J. Infect. Dis..

[54] Kocazeybek B (2017). Evaluation of Adenovirus-36 (Ad-36) antibody seropositivity and adipokine levels in obese children. Microb. Pathog..

[55] Berger PK (2014). Association of adenovirus 36 infection with adiposity and inflammatory-related markers in children. J. Clin. Endocrinol. Metab..

[56] Kuczmarski RJ (2002). 2000 CDC growth charts for the United States: Methods and development. Vital Health Stat..

[57] Freedman DS, Mei Z, Srinivasan SR, Berenson GS, Dietz WH (2007). Cardiovascular risk factors and excess adiposity among overweight children and adolescents: The Bogalusa heart study. J. Pediatr..

[58] Rizzo AC (2013). Metabolic syndrome risk factors in overweight, obese, and extremely obese Brazilian adolescents. Nutr. J..

[59] Fernández JR, Redden DT, Pietrobelli A, Allison DB (2004). Waist circumference percentiles in nationally representative samples of African-American, European-American, and Mexican-American children and adolescents. J. Pediatr..

[60] Sinaiko AR (2006). Influence of insulin resistance and body mass index at age 13 on systolic blood pressure, triglycerides, and high-density lipoprotein cholesterol at age 19. Hypertension.

[61] Matthews DR (1985). Homeostasis model assessment: Insulin resistance and beta-cell function from fasting plasma glucose and insulin concentrations in man. Diabetologia.

[62] Katz A (2000). Quantitative insulin sensitivity check index: A simple, accurate method for assessing insulin sensitivity in humans. J. Clin. Endocrinol. Metab..

